# Exploring the effects of stationary camera spots on inferences drawn from real-time crash severity models

**DOI:** 10.1038/s41598-022-24102-y

**Published:** 2022-11-25

**Authors:** Amirhossein Abdi, Seyedehsan Seyedabrishami, Carlos Llorca, Ana Tsui Moreno

**Affiliations:** 1 Faculty of Civil and Environmental Engineering, Tarbiat Modares University, P.O. Box 14115-397, Tehran, Iran; 2 Technical University of Munich, Munich, Germany

**Keywords:** Civil engineering, Computer science

## Abstract

This study combined crash reports, land use, real-time traffic, and weather data to form an integrated database to analyze the severity of crashes taking place on rural highways. As the traffic cameras are placed at fixed locations, there is a wide range of measured distances between crashes and the selected nearest camera for extracting traffic variables. This may change the significance of traffic variables. For the first time, spacing was introduced as the distance around the detectors in which traffic characteristics are inferred to crashes. Classification and Regression Tree (CART) was employed as an interpretable tool to explore how spacing affects model performance and the significance of traffic variables. Twelve spacing scenarios from 250 to 3000 m were evaluated. Except for short spacings suffering from the low sample size issue, each model has a good predictive performance based on overall accuracy and F_2_ score in the 1000–3000 m spacings. In this range, three dominant rules emerged: (1) high deviations of speed on the roads surrounded by wastelands are associated with severe crashes; (2) faded markings in residential zones increase the likelihood of severe outcomes; (3) installation of barriers decrease the probability of severe crashes. Comparing the Variable Importance Measure (VIM) reveals that the total importance of traffic variables reduces as the spacing increases. Also, results indicate that average speed is significant until 1750 m; but speed deviation, traffic flow, and percent of heavy vehicles are more stable variables for further spacings. In conclusion, for the first time, spacing scenarios were evaluated systematically and proved that they have a remarkable impact on the significance of variables. This novel research provides guidance not only on the spacing but also on which real-time traffic variables have a greater impact on crash severity, along with design, land use, and environmental variables.

## Introduction

The road network of Iran consists of 206,974 rural roads and 43,946 main roads^1^. According to the world health statistics, the estimated road traffic fatality rate for 2019 was 21.47 deaths per 100,000 persons in Iran. Moreover, road deaths have increased among low- and middle-income countries^2^. Consequently, conducting a crash severity analysis on rural roads of low- and middle-income countries is of high importance, and safety professionals could benefit from the results of this paper to explore influential factors and decrease severe crashes occurring in these less-investigated segments.

As mentioned, one of the main objectives of this research is to identify the key factors contributing to crash severity on rural roads. Various behavioral, environmental, geometric, and vehicle-related factors influence crash injury severity^3^. Variables relating to these factors could be obtained from numerous data sources. This study conducted crash severity analysis for rural highways of Khorasan Razavi province, located in northeastern Iran. Multiple datasets consisting of traditional crash data, land use characteristics obtained from base maps, weather factors obtained from synoptic weather stations, and real-time traffic variables captured by traffic cameras were utilized to form an integrated database.

Because of relying on witnesses’ statements and rounding numbers, most crash documented times are inaccurate at minute-level^4^. The impact of incorrect times possibly harms studies that utilize real-time traffic variables before the crash. A reasonable pre-crash time interval that compensates for errors has been introduced concerning this problem. Along with inaccurate documented times, a crash could occur anywhere on the road network; meanwhile, the density of detectors varies for different road segments and includes them at fixed locations. These lead to varying distances between crashes and the nearest camera to their location^3,5^. Unlike previous studies, which only reported detector layout without considering these issues, the main novelty of this research is introducing spacing scenarios as the area covered by each detector in the modeling process. While a larger scenario will improve coverage and increase the number of crashes with assigned traffic characteristics, it may also affect the exactness and importance of associated pre-crash traffic variables to the crash location. This novel approach intends to provide knowledge and direction on including different coverage scenarios in the modeling procedure. Also, it benefits safety monitoring systems with the idea of having numerous online prediction tools for different coverage scenarios rather than dismissing different detector-to-crash distances.

Crash severities were classified into two severe (serious injury and fatal crashes) and non-severe (property damage only and light injury crashes) classes. Classification and Regression Tree (CART), one of the most interpretable and widely used data mining algorithms, was employed to identify significant factors and key patterns determining crash severity for each spacing scenario. In addition, the prediction power of models and variable importance measures have been computed to investigate the impact of spacing scenarios.

## Background

The real-time crash prediction model (RTCPM) is a popular topic developed to serve various purposes in road safety research. Unlike conventional models containing driver and vehicle-related factors, RTCPMs mainly rely on readily available variables like traffic variables collected by detectors preceding a crash^3,6^. Crash severity prediction and exploring related contributing factors are one of the main objectives of constructing RTCPMs. Most of the studies in this realm have considered interstate freeways, expressways, and arterials as their study areas^7^. Therefore, other facilities, including rural roads, have not received proper attention. This study has been conducted to narrow the existing gap by considering the severity of crashes occurring on rural roads.

Regarding the real-time traffic variables, different statistics of speed, flow, and occupancy recorded by different sensors with respect to the crash location and time are the core part of the RTCPMs^7^. The considered traffic flow variables and associated results for some crash severity studies are summarized in Table 1.

**Table 1:** Traffic flow variables and associated results in crash severity studies.

Study	Authors (year)	Considered traffic flow variable	Results
^8^	Yu and Abdel-Aty (2014)	Standard deviation of speed	standard deviation of speed was significant with a positive sign indicating that large variations increase the likelihood of severe crashes
^9^	Yu and Abdel-Aty (2014)	Standard deviation of speed	Large variation of speed is associated with severe outcomes
^10^	Choudhary et al. (2018)	Total flow Average Speed Between lanes speed variation Within lane speed variation	The within lane speed variance is associated with high rates of killed or seriously injured casualties. At high traffic flows, fatal and injury crash rates increase with increasing within lane speed variance. At high speeds, fatal and injury crash rates increase with increase in between lane speed variance
^11^	Zeng et al. (2019)	The percentage of vehicles in five vehicle classes	With a greater portion of light trucks, minivans, and minibusses, fatal and injury crashes are more likely to occur. A greater number of large trucks, buses, and trailers increase the chance of slight and severe injuries

In addition to the traffic variables, numerous safety studies have used an extensive range of roadway and environmental characteristics. Table 2 presents these factors in some of the previous safety studies chronologically. Nonetheless, to the best of our knowledge, previous literature rarely investigated land use variables. Because of the geographical vastness of the present study, land use factor is considered to substitute other variables such as speed limit and density of accesses, which are hard to collect for large study areas. Also, its relationship was examined with the severity of crashes taking place on rural roads.

**Table 2:** Studies with roadway and environmental factors.

Study	Authors (year)	Roadway characteristics	Environmental characteristics
^12^	Abdel-Aty and Abdalla (2004)	Curvature, existence of barriers, median width and type, pavement surface, ramps, shoulder width and type	–
^13^	Kashani and Mohaymany (2011)	Shoulder type	Lighting conditions, Pavement conditions (function of precipitation), Weather conditions
^14^	Jung et al. (2016)	Curvature, lane width, number of lanes, pavement surface, shoulder width, speed limit	Lighting conditions, rainfall intensity, temperature, wind direction, wind speed
^15^	Shi et al. (2018)	Curvature, existence of barriers, pavement surface, road markings, traffic signs	–
^16^	Yasmin et al. (2018)	Median width, number of lanes, ramps, shoulder width, speed limit	Weather conditions
^17^	Ali et al. (2019)	–	Pavement conditions, visibility conditions, weather conditions
^18^	Wang and Prato (2019)	Curvature, gradient	Pavement conditions, weather conditions

From a methodological standpoint, statistical and artificial intelligence approaches have been employed in the existing literature. Among statistical methods, different types of logit models have been widely used by researchers^5,19–22^. Recent studies overwhelmingly relied on Bayesian inference in parameter estimation for RTCPMs^23,24^. Moreover, artificial intelligence methods as non-parametric techniques have been preferred by researchers to capture nonlinearities that exist between outcomes and explanatory variables. Different forms of neural networks^25–27^, Bayesian networks^5,28^, support vector machines^25,27^, and decision trees^24,29–31^ are frequently proposed models among non-parametric techniques.

Regarding decision trees, researchers have used decision tree models to explore and interpret the determinants of injuries for rural roads^13^. Recently, it has been employed to examine interactive effects between multiple factors on the severity of freeway crashes^29^. Decision trees and tree-based algorithms have the advantage of ranking and selecting significant variables intrinsically, making them effective safety research methods^7^. By considering the fact that combinations of multiple factors contribute to severity outcome, this study utilized Classification and Regression Tree (CART) as a decision tree algorithm with the advantage of capturing interactive relationships and producing graphical representations making interpretability easy to understand. With the benefit of this algorithm, the large number of variables, which is a consequence of combining multiple datasets, is not a problem anymore, and the algorithm provides beneficial results by choosing a few key variables^32^.

## Data preparation

**Figure 1:**
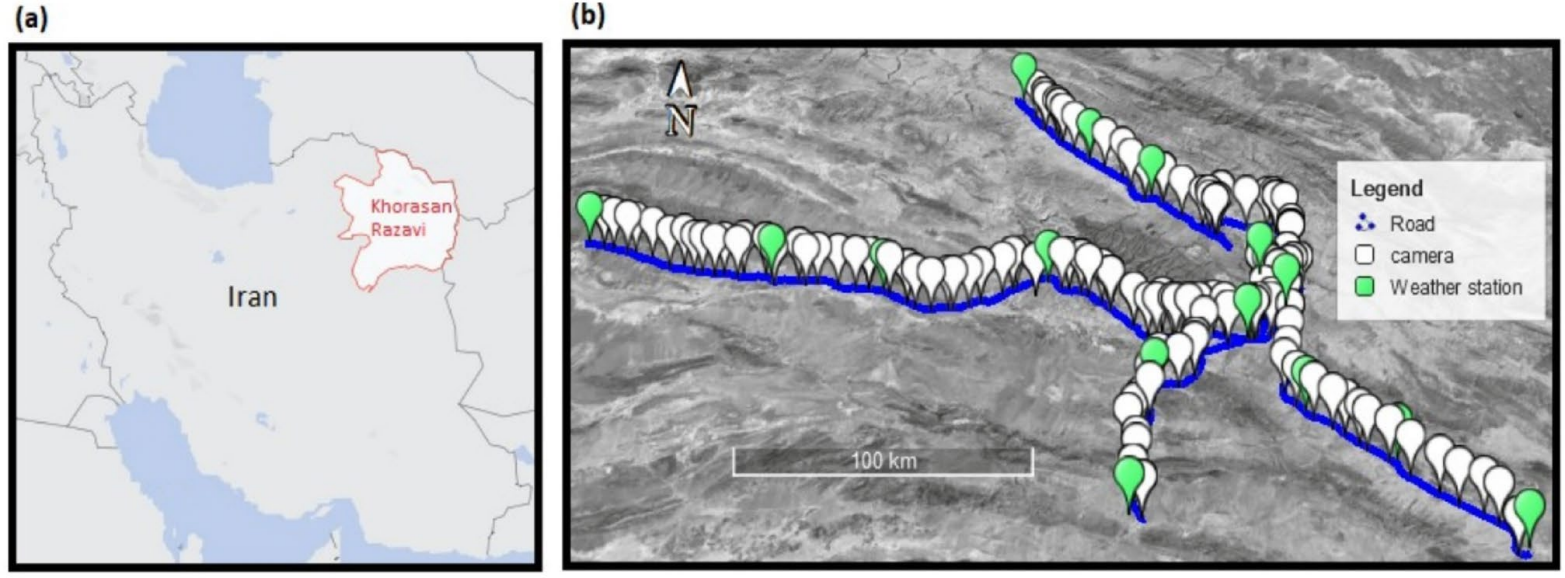
(**a**) Illustration of the study area. The map is drawn using QGIS version 2.18.4 (https://qgis.org/en/site/), (**b**) layout of camera and weather stations on the selected highways. The satellite image and details were prepared in Google Earth Pro version 7.3.4.8642 (https://www.google.com/earth/versions/).

In order to form an integrated database, four datasets were combined; (1) crash dataset for rural highways in the Khorasan Razavi province for 6 years (from 2015 to 2020), including roadway and geometry characteristics provided by the Road Maintenance and Transportation Organization (RMTO), (2) land use characteristics represented by base maps from Open Street Map (OSM), (3) real-time weather data collected by 15 synoptic weather stations along the study area (from 2015 to 2020), and (4) real-time traffic data recorded by 131 traffic cameras with an average spacing of 5.52 km between stations along rural highways (from 2015 to 2020). Figure 1 illustrates the study area and the layout of cameras and weather stations. Rural highways in this area present low curvature degrees and level terrain.

**Figure 2:**
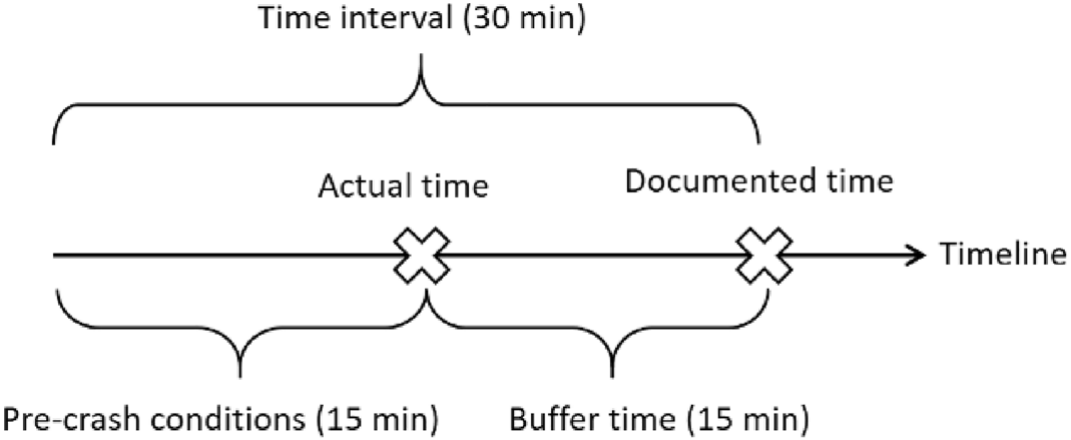
Parts of the specified time interval.

Most documented times suffer from inaccuracies at the minute level. The distribution of recorded crash times indicates that 53.21% of rural crashes have documented times that minute indication (MM) ended with 00 or 30. If it is assumed that crash time is usually recorded at a later time with respect to the actual time, the specified time interval before the documented time consists of two parts; a buffer time that compensates for errors and the remaining part which is representative of the traffic conditions prior to the crash^21,33,34^. Applying an appropriate buffer time could be an effective remedy for errors in documented crash time. Because of the high portion of rounding minutes to the nearest half hour, a 15 min buffer time has been applied in this study. Figure 2 shows the components of the specified time interval.

**Figure 3:**
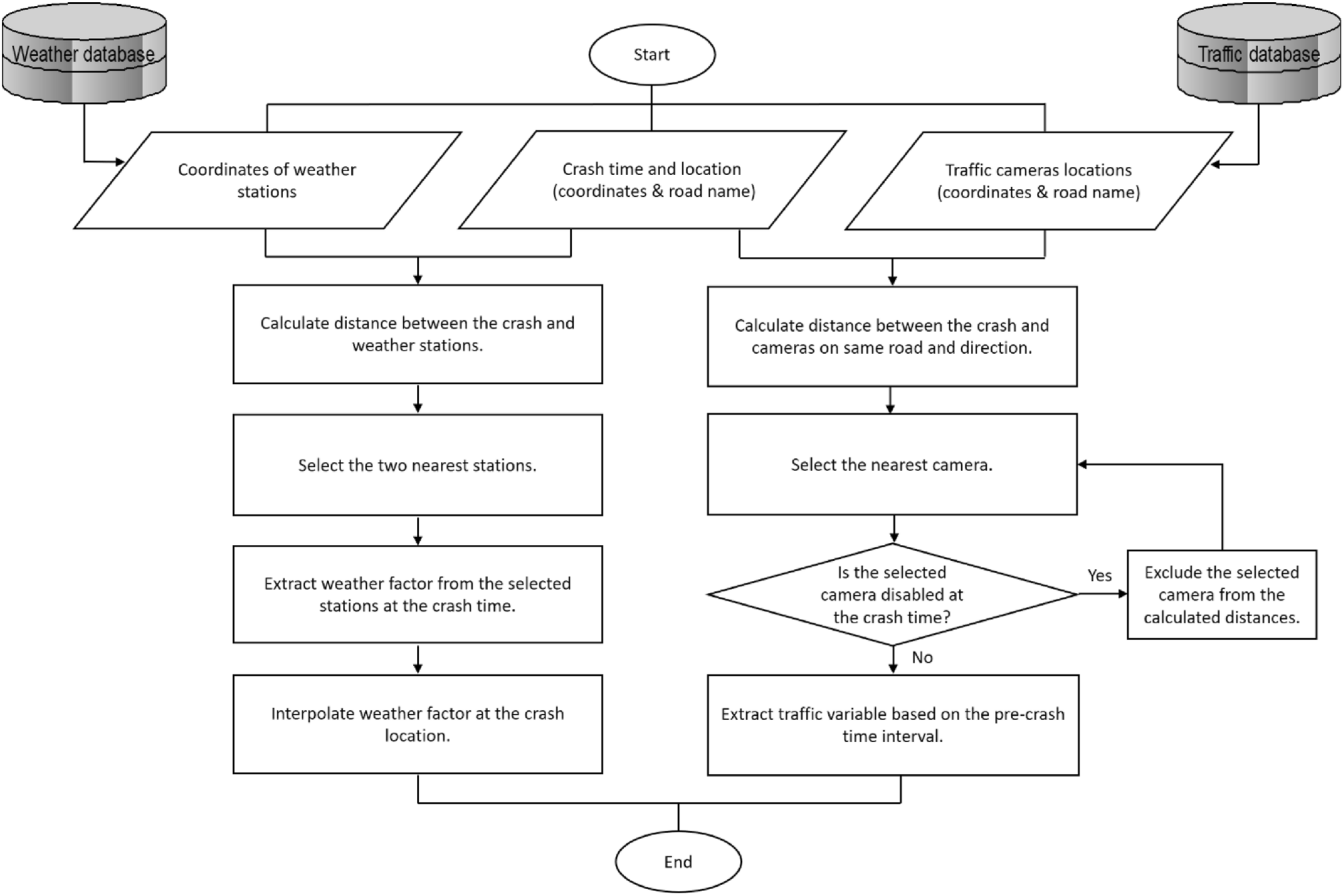
Flowchart of fusing traffic and weather data with crash data.

Figure 3 represents a flowchart for extracting traffic and weather variables from related datasets. The nearest available camera to each case is employed to extract traffic variables. To the traffic cameras, each vehicle is just a data point that includes speed, lane, type, date, and time of the vehicle passing in front of the camera. Inevitable errors in reported coordinates may locate some records in wrong directions or even lands adjacent to the road. According to this point, assigning a crash to the nearest camera only based on geographical coordinates would be misleading. In this respect, utilizing the road name and direction (crash address) along with coordinates has been recommended. The definition of traffic variables that were utilized for this research are as follows:

- Average speed: within the pre-crash conditions, the mean of vehicles' speeds passing in front of the selected camera is considered average speed.
- Logarithmic transformation of flow: during the pre-crash time interval, the logarithmic transformation of the total number of vehicles counted by the nearest camera is the logarithmic transformation of flow.
- Percentage of heavy vehicles: It is computed by the proportion of heavy vehicles out of the total traffic count for the pre-crash conditions at the nearest camera.
- Standard deviation of speed: The standard deviation of speed refers to speed deviation across all the vehicles present at the location of the nearest camera for pre-crash conditions.

As shown in Fig. 3, for each crash, weather factors, including the amount of precipitation, visibility, temperature, and weather categories, were extracted based on the documented crash time from the two nearest stations; then, by means of Inverse Distance Weighting (IDW) interpolation, the corresponding weather factor was calculated at the location of the crash. This simple technique uses the measured climatic values from the nearest stations; then, It weights them based on their distance to the crash location^35^. The closer the station, the more weight has its related climatic factor. Equation (1) represents the IDW technique for calculating weather factor $w$ from the two nearest stations:

1 $$w=\frac{{w}_{1}\times \frac{1}{{d}_{1}}+{w}_{2}\times \frac{1}{{d}_{2}}}{\frac{1}{{d}_{1}}+\frac{1}{{d}_{2}}}$$

where ${w}_{1}$ and ${w}_{2}$ are weather factors for the nearest and the second nearest stations, respectively. Also, ${d}_{1}$ and ${d}_{2}$ are measured distances to the nearest and the second closest stations, respectively.

After combining traffic and weather data with crash data, land use characteristics were obtained through base maps at the crash location and address. In this regard, a buffer area is generated around each crash in the network, and the percentage of area occupied by different land use types is calculated. Finally, the most dominant type is assumed to be the crash's land use type.

The resulting integrated database contains 126 variables. Table 3 summarizes the descriptive statistics for the variables shown to be significant in the models.

**Table 3:** Summary of variables descriptive statistics.

Variables	Description	Mean	Std. dev	Min	Max
**Dependent variables**					
Severity	1 if severe crashes; 0 if non-severe crashes	0.28	0.45	0	1
**Independent variables**					
**Land use**					
Industrial area	1 if crash occurred in industrial area; 0 otherwise	0.03	0.17	0	1
Residential zone	1 if crash occurred in residential zone; 0 otherwise	0.21	0.41	0	1
Wasteland	1 if crash occurred next to wasteland; 0 otherwise	0.35	0.48	0	1
**Roadway characteristics**					
Faded markings	1 if road markings were faded ; 0 otherwise	0.09	0.29	0	1
Installed barriers	1 if road barriers were installed ; 0 otherwise	0.22	0.41	0	1
Shoulder type	1 if shoulder was paved; 0 if shoulder was unpaved	0.37	0.48	0	1
**Traffic variables**					
Avg speed	Average speed (km/h)	84.16	14.87	36.92	128.37
Log of flow	Logarithmic transformation of flow	2.24	0.60	0	3.32
Pct heavy vehicles	Percentage of heavy vehicles	4.64	9.03	0	42.86
SD speed	Standard deviation of speed (km/h)	7.11	6.08	0	28.48
**Weather variables**					
Temperature	Temperature (degrees of Celsius)	16.27	8.31	− 6.49	40.43

## Methodology

### Decision tree

Classification is the task of identifying a model that assigns input data with a class label. The model is initially generated based on the training dataset, a set of observations with known target variables. Finally, the generated model will make predictions on an unseen dataset known as the testing dataset^36^. Classification and Regression Tree (CART) is one of the most important methods in data mining that refers to decision tree algorithm. CART can be employed for nominal target variables like crash severity (classification tree) and also continuous ones (regression tree)^37^.

**Figure 4:**
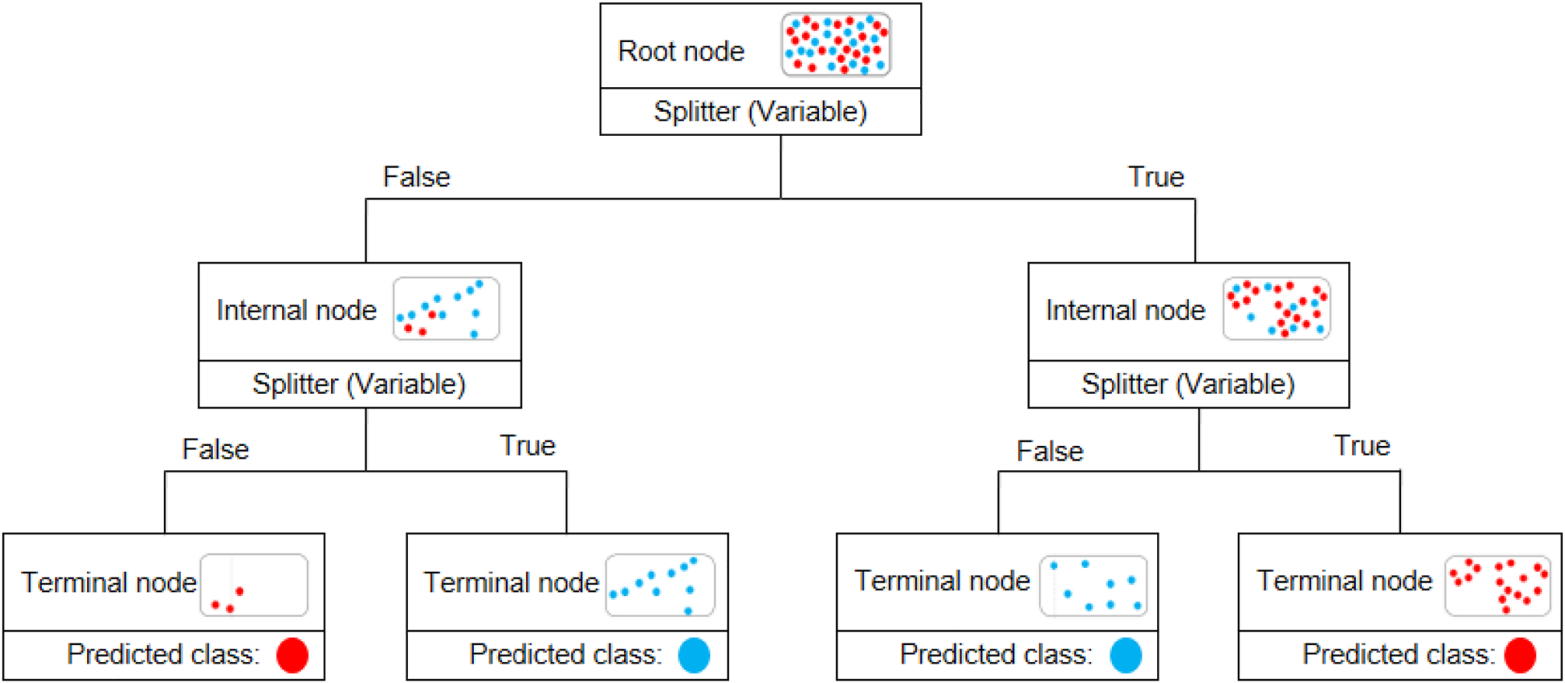
The fundamental principles of decision tree.

Figure 4 represents the fundamental principles of the decision tree. The structure of every tree consists of three types of nodes; (1) a root node, (2) internal nodes, and (3) terminal nodes. A root node is always the first node in the path that all the inputs pass through. The root node is divided into further sub-nodes by an independent variable or splitter. Splitting is the process in which conditional statements (if–then statements) appear to create relatively pure or homogenous sub-nodes. The process is an iterative one until the greatest possible homogeneity is achieved. Every path in the tree ends with a terminal or leaf node, which does not split any further. Nodes between the root and the leaf nodes are identified as internal nodes.

**Figure 5:**
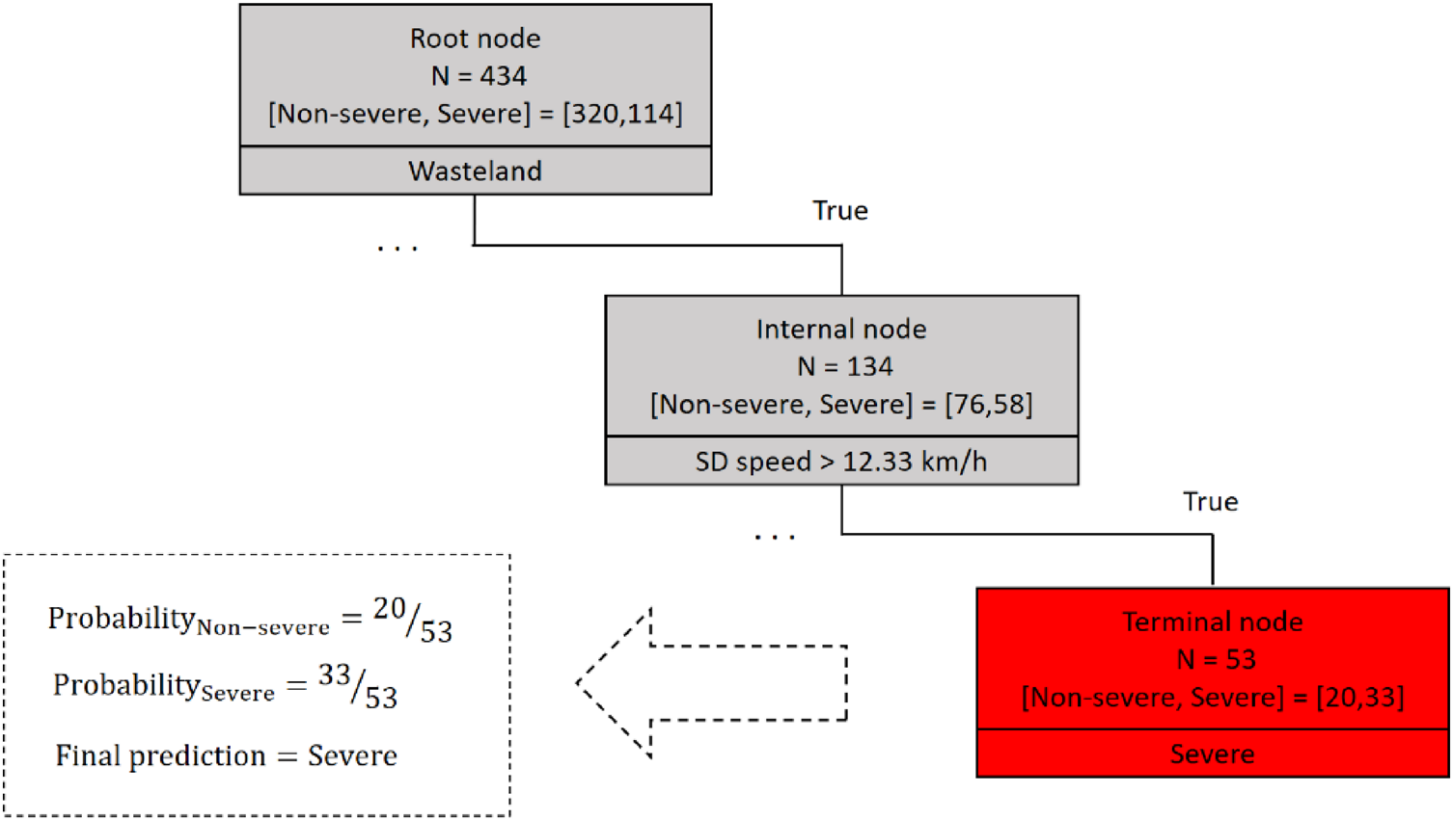
Extracting a rule from the related decision path.

For making interpretations and extracting rules from the graphical representation of the decision tree, it is required to read associated conditions for a particular path. Each decision path in this algorithm starts from the root node and ends with a specific terminal node. Figure 5 illustrates a decision path with related conditional statements. According to this path, if wastelands surround the crash location (wasteland = true) and if the standard deviation of speed for the crash is greater than 12.33 km/h (SD speed > 12.33 km/h), the probability of a severe crash is 33/53, which is greater than the probability of a non-severe outcome (20/53). Therefore, the associated rule of this path signifies that speed deviation greater than 12.33 km/h in wasteland areas could lead to severe crashes.

There are different rules in the execution of the decision trees. In the realm of this article, the Gini index will be explained. The classic CART algorithm uses the Gini index, also known as Gini impurity, to construct the tree. Suppose a tree with $m\in \left\{\mathrm{1,2},\dots ,M\right\}$ nodes, the Gini index of node $m$ is calculated as Eq. (2).

2 $$Gini(m)=1-\sum_{i=1}^{S}{P}_{im}^{2}$$

where ${P}_{im}$ is the probability of an observation being classified for a specified severity of $i\in \left\{\mathrm{1,2},\dots ,S\right\}$ in node $m$. ${P}_{im}$ is calculated as Eq. (3).

3 $${P}_{im}=\frac{\frac{\pi (i){N}_{i}(m)}{{N}_{i}}}{\sum_{i=1}^{s}\frac{\pi (i){N}_{i}(m)}{{N}_{i}}}$$

where $\pi (i)$ is the prior probability for severity $i$ (proportion of every class in population), ${N}_{i}(m)$ is the number of observations in severity $i$ of node $m$, and ${N}_{i}$ is the number of observations of severity $i$ in the dataset.

Gini index takes values between zero and one. If most observations fall into the same class (crash severity) in a node, Gini will have small values. Conversely, if there is an equal number of observations across all classes in a node, the Gini will be maximized. The splitting process will be terminated when the smallest possible Gini index is gained.

Decision trees are infamous for overfitting, which results in overly large trees being too fit to the training dataset but high misclassified predictions on the testing dataset. In this regard, the completely grown tree is pruned by eliminating some of the nodes. Minimal cost-complexity pruning is an algorithm that takes a sub-tree from the completely grown tree by minimizing the $Tree\ Score$ in Eq. (4).

4 $$Tree\ Score=R\left(T\right)+\alpha \left|T\right|$$

where $T$ is a pruned sub-tree, $\left|T\right|$ is the number of leaf nodes in $T$, $R(T)$ is the misclassification error of the tree $T$, and $\alpha$ is the complexity parameter that penalizes the complex tree with more leaf nodes. Equation (4) is calculated repeatedly for different alpha values from zero to one, resulting in a tree sequence. The optimal pruned tree is the sub-tree with the least misclassification error based on a simple split-sample validation. Further information on the CART algorithm is available at^37^.

Providing insights is one of the main objectives of a modeling project. To this end, the Variable Importance Measure (VIM) represents the contribution of each variable with respect to others in a classification or regression task. The $VIM$ of an independent variable ${x}_{j}$ is calculated as Eq. (5).

5 $$VIM\left({x}_{j}\right)=\sum_{m=1}^{M}\frac{{N}_{m}}{N}\Delta Gini\left(S\left({x}_{j},m\right)\right)$$

$M$ denotes the total number of nodes, $N$ and ${N}_{m}$ represent the total number of observations in the dataset and the number of observations in the parent node $m$, respectively. $\Delta Gini\left(S\left({x}_{j},m\right)\right)$ stands for the Gini reduction at parent node $m$ which is achieved by splitter ${x}_{j}$. $\Delta Gini\left(S\left({x}_{j},m\right)\right)$ is defined as Eq. (6).

6 $$\Delta Gini\left(S\left({x}_{j},m\right)\right)=Gini\left(m\right)-\frac{{N}_{mL}}{{N}_{m}}Gini\left({m}_{L}\right)-\frac{{N}_{mR}}{{N}_{m}}Gini({m}_{R})$$

where ${N}_{mL}$ and ${N}_{mR}$ stand for the number of observations in the child nodes ${m}_{L}$ and ${m}_{R}$, respectively. Higher VIMs indicate the great importance of the corresponding variable^37^.

### Handling imbalanced crash severity data for developing trees

Crash severity datasets have unequal class distribution with fatal and serious injury labels in minority classes^38^. Class imbalance is prevalent in many real-world classification tasks and will bias the prediction model towards the majority class^39^. To address this problem, converting different severities to a binary prediction problem reduces the class imbalance. This approach has been suggested in several previous studies^8,9,13,29,40^. Furthermore, adjusting prior probabilities in Eq. (3) produces trees with terminal nodes predicting minority class^13^.

### Model evaluation metrics

Model evaluation metrics measure the performance of a trained model on an unseen dataset and tell how well the model generalizes. Numerous metrics have been introduced for performance evaluation in different applications. Taking proper metrics is a critical point in the evaluation process.

Several studies used confusion matrix related metrics such as accuracy, precision, recall and ${\mathrm{F}}_{1}$ to evaluate classification models^38,39,41^. A confusion matrix is a two-dimensional table with columns representing the predicted class instances and rows indicating the actual class for a binary classification problem. Table 4 shows the confusion matrix, wherein the true positives (TP) and the true negatives (TN) are correctly predicted classes, and the false positives (FP) and the false negatives (FN) are incorrectly classified instances.

**Table 4:** Confusion matrix.

	Predicted positive class	Predicted negative class
Actual positive class	True Positive (TP)	False Negative (FN)
Actual negative class	False Positive (FP)	True Negative (TN)

The definition of some classification metrics is as follows^38^:

- Accuracy is defined as the ratio of true predictions to the total number of inputs. Accuracy is a necessary metric to report. It indicates whether the model's overall performance is satisfying. But when the cost of misclassification of the classes varies, other metrics should be considered as well. This is mostly because of the serious social and economic costs of severe crashes. Therefore, considering a specific metric that could evaluate the model with respect to predicting severe crashes could be beneficial. This metric will be introduced in the final part of this section.
  7 $$Accuracy=\frac{TP+TN}{TP+TN+FP+FN}$$
- Precision specifies the exactness of a model by calculating the ratio of correctly classified classes among all predicted classes. Poor precision is a sign of several false positives.
  8 $$Precision=\frac{TP}{TP+FP}$$
- Recall determines the completeness of a classifier by calculating the ratio of truly predicted positives. Poor recall indicates many false negatives.
  9 $$Recall=\frac{TP}{TP+FN}$$
- ${F}_{\beta }$ is a measure combining both precision and recall using the beta parameter for weighting the balance between them. When maximizing recall is the priority, a beta value of $\beta >1$ would be preferred. On the contrary, beta values smaller than 1 prioritize maximizing precision. For $\beta =1$, ${F}_{\beta }$ gives equal weight to both precision and recall^41^.
  10 $${F}_{\beta }=\left(1+{\beta }^{2}\right)\times \frac{Precision\times Recall}{{\beta }^{2}\times Precision+Recall}$$
  As mentioned in the definition of accuracy, it is vital to reduce the number of severe crashes which were misclassified as non-severe cases; in this regard, calculating the ${F}_{2}$ measure (${F}_{\beta }$ with $\beta =2$) for severe crashes as a metric that gives more weight to recall would be informative. Higher values of ${F}_{2}$ signify that model misclassified fewer severe cases as non-severe outcomes.

## Modeling results and discussion

### Spacing scenarios

**Figure 6:**
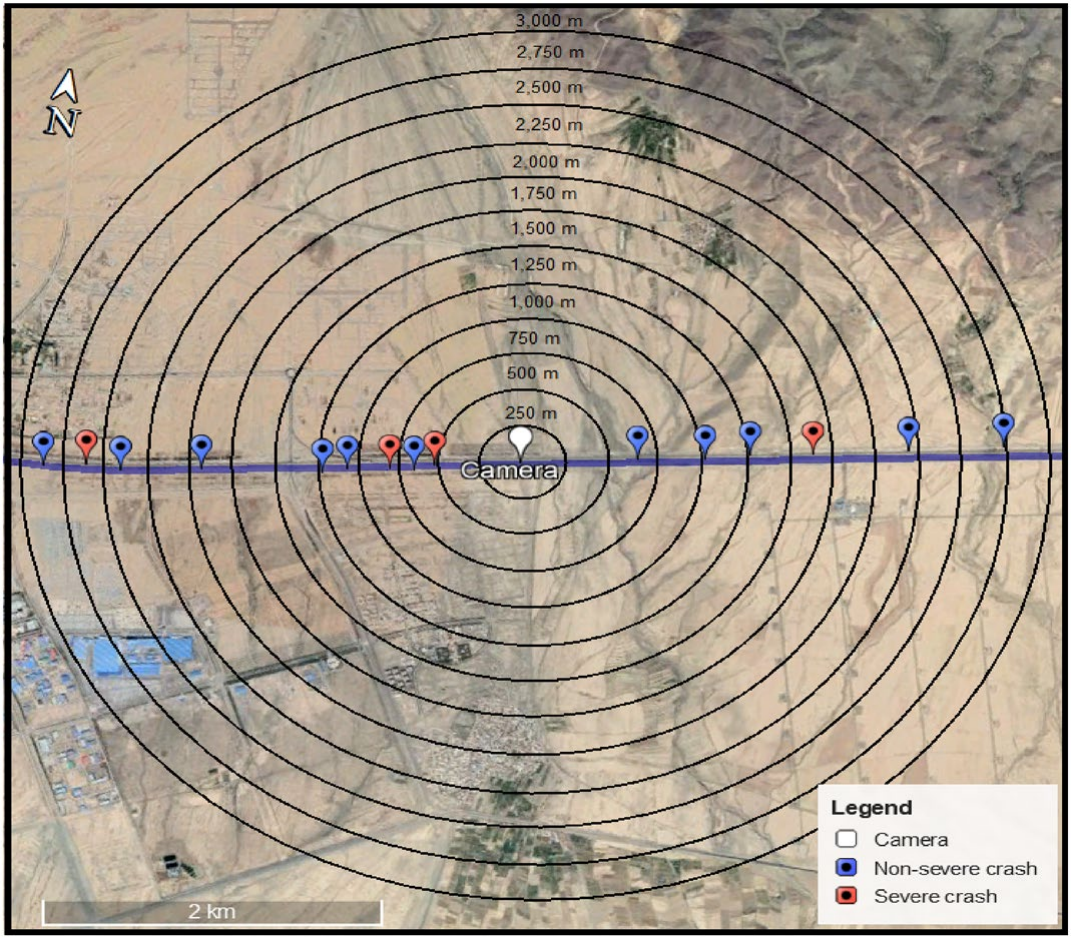
Illustration of spacing scenarios.

As mentioned before, each crash has been assigned to the nearest available camera to extract traffic variables. Calculated distances between crash locations and their nearest camera vary from 6 m to approximately 3000 m. Instead of considering all observations as a unified modeling input and reporting detector layout like previous studies^19,42–44^, multiple spacing scenarios were introduced. Each spacing scenario considers a threshold distance which is the maximum distance for assigning a record to the nearest camera. For instance, the 1000 m spacing is defined as a scenario in which a camera is located less than 1000 m away from crashes. Simply put, each spacing scenario represents an area around the camera that is covered by that camera. Twelve spacings with a fixed step size of 250 m from 250 to 3000 m were introduced. Figure 6 shows these spacings with associated crashes for one of the cameras in Fig. 1b. For each spacing, Crashes at a distance lower than the spacing were assigned to the camera and included in the analysis, but the crashes outside of the circle were discarded.

Reporting overall accuracy as a performance measure is necessary but not sufficient due to the imbalanced nature of severities and the high costs of severe crashes, which may lead to erroneous conclusions^41^. Therefore, F_2_ score, which prioritizes recall, is calculated for severe crashes.

**Figure 7:**
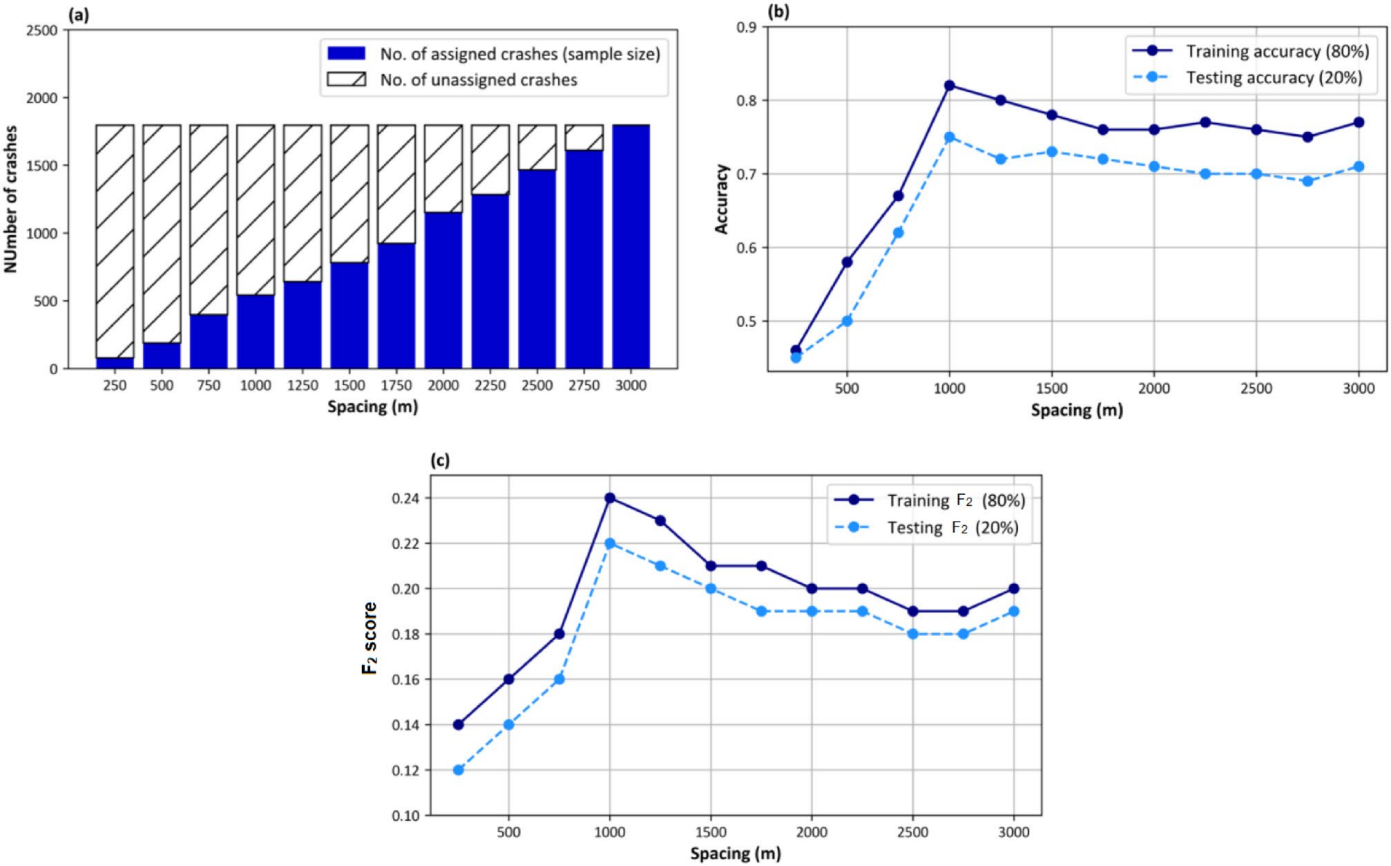
(**a**) Specification of each spacing scenario, (**b**) accuracy for each spacing, (**c**) F_2_ score of severe crashes for each spacing.

Figure 7 presents proposed scenarios and their related sample size and performance measures. It can be concluded that when the spacing is small, the performance of the models is poor due to the small sample size. The sample size would increase by increasing spacing size, and therefore fewer crashes remain unassigned. It can be seen that each model in the 1000–3000 m range has satisfactorily good metrics. Therefore, we can conclude that as far as low sample size is not the problem, high predictive power can be reached in different coverage scenarios.

Regarding the 1000–3000 m range, we will analyze and interpret the resulting graphical representations for 1000, 2000, and 3000 m decision trees in the following three sections. Finally, we will examine the VIM for this range in the last section of the modeling results.

### Decision tree of 1000 m spacing

**Figure 8:**
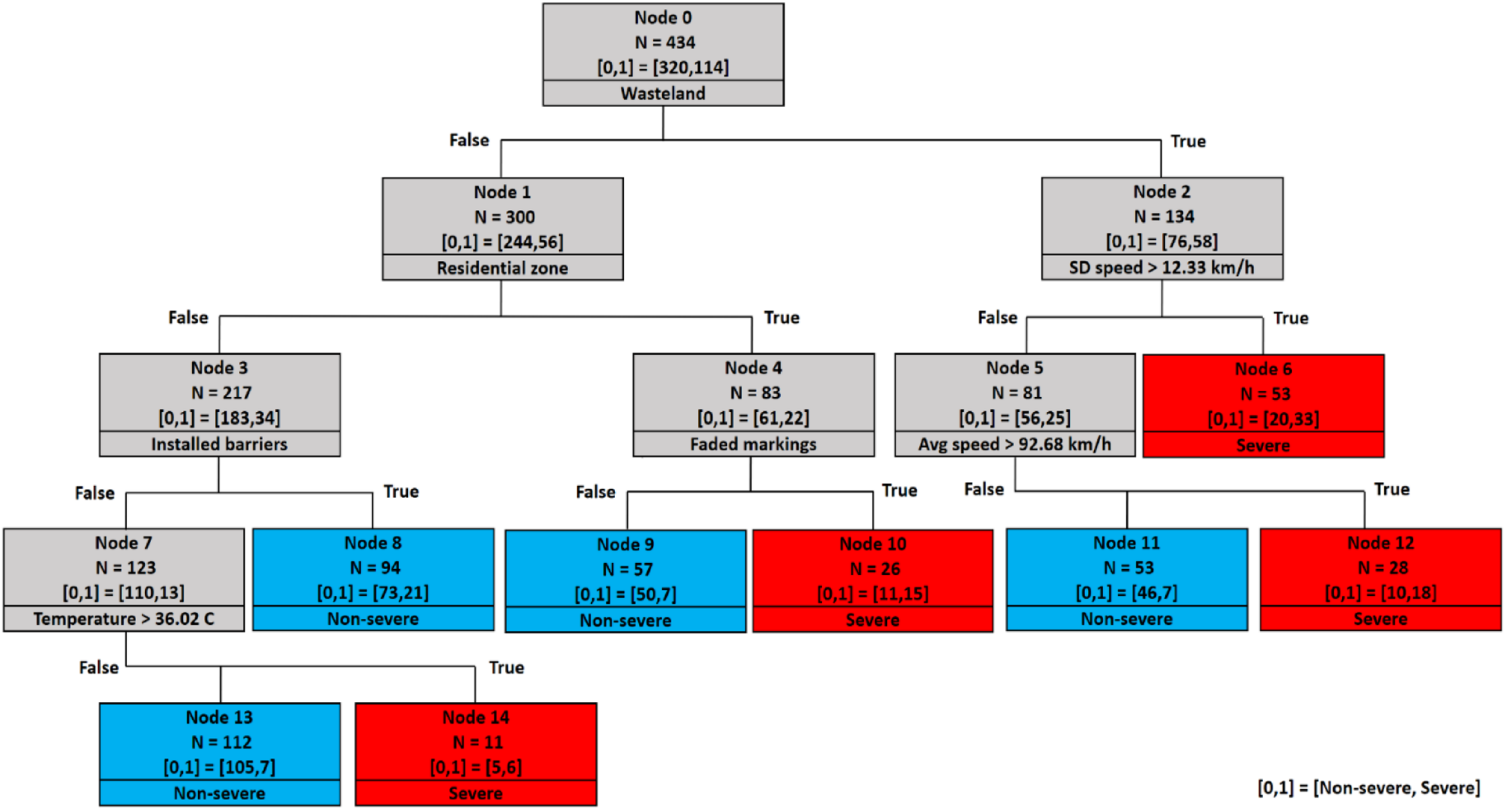
Decision tree of 1000 m spacing scenario.

The first scenario includes crashes with a maximum 1000 m distance from the nearest camera. The decision tree of the 1000 m spacing scenario is displayed in Fig. 8. On the top of the tree, node 0, which is the root node, is divided based on the wasteland variable. It implies that the wasteland variable is the most significant variable in predicting the severity of rural roads crashes.

For locations across wastelands (right side of the tree), the next significant variable in this branch is standard deviation of speed. Specifically, standard deviations of speed greater than 12.33 km/h lead to severe outcomes (leaf node 6). For standard deviations of speed lower than 12.33 km/h, average speed is checked next (node 5). Splitting this node reveals that severe crashes are possible if average speed passes the threshold of 92.68 km/h, speed deviation less than or equal to 12.33 km/h, and the road is located within wastelands (terminal node 12). As seen, there are interactive effects between speed deviation and average speed, which is in line with the study that showed higher speeds coupled with variations in speed increase the risk of injury and fatal crashes^10^.

For locations not in wastelands, the tree is separated based on the residential land use variable. Segments with faded markings in residential areas increase the likelihood of severe crashes (terminal node 10). On the other hand, for non-residential areas which are not located in wastelands, the next significant variable is the presence of safety barriers (node 3). Splitting node 3 reveals that the presence of safety barriers decreases the likelihood of severe crashes (leaf node 8). Finally, severe crashes are more likely to occur at temperatures greater than 36.02 degrees Celsius when no safety barriers are installed (leaf node 14). These conditions generally correspond to extremely hot summer days. This result confirms studies concluded that warm seasons^45^ and extreme temperatures^46^ are correlated with severe crashes and unsafe conditions.

### Decision tree of 2000 m spacing

**Figure 9:**
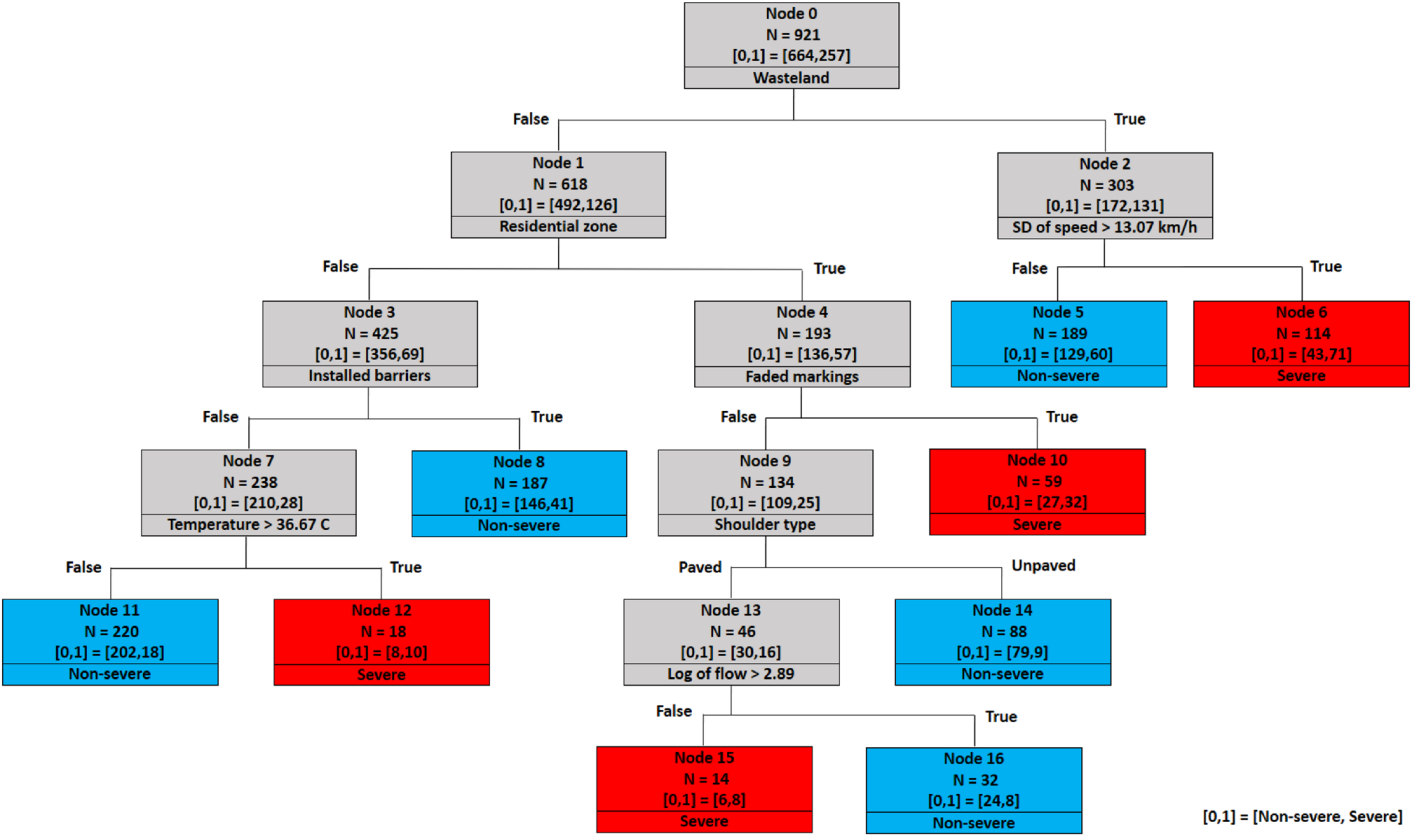
Decision tree of 2000 m spacing scenario.

This section interprets the decision tree of the 2000 m scenario shown in Fig. 9. The root node is separated by the wasteland variable, which emphasizes the importance of this variable similar to the previous model.

For wasteland roads, Similar to the 1000 m scenario, high standard deviations of speed (> 13.07 km/h) increase the likelihood of severe crashes (leaf node 6). However, the average speed is not significant for this spacing compared to the 1000 m spacing. This implies that the increased spacing reduced the accuracy on average speed and faded its significance.

Residential land indicator is the next determinant when the crash is not located in wastelands. Like the previous scenario, in the presence of faded markings in residential areas, severe outcomes are more likely (leaf node 10). In addition to the faded marking indicator, shoulder type and logarithm of flow were identified as significant variables for residential areas in this scenario. Rules associated with these two new variables imply that unpaved shoulders decrease the chance of severe crashes (terminal node 14). Besides, paved shoulders and logarithm of flow show an interactive effect on crash severity; When the shoulder is paved, low traffic flows (logarithm of flow $\le$ 2.89) could result in occurring severe crashes (leaf nodes 15). Previous studies have pointed to the road shoulder as a determinant factor for rural road crashes^47,48^. This study adds to the literature by this point that the impact of paved shoulders on crash severities is interactive with the amount of flow.

Like the tree of 1000 m spacing, for records not located in wastelands and residential areas, the presence of safety barriers is most likely linked to non-severe outcomes (terminal node 8); Similarly, at temperatures greater than 36.67 degrees of Celsius, severe crashes are more likely to occur (leaf node 12).

### Decision tree of 3000 m spacing

**Figure 10:**
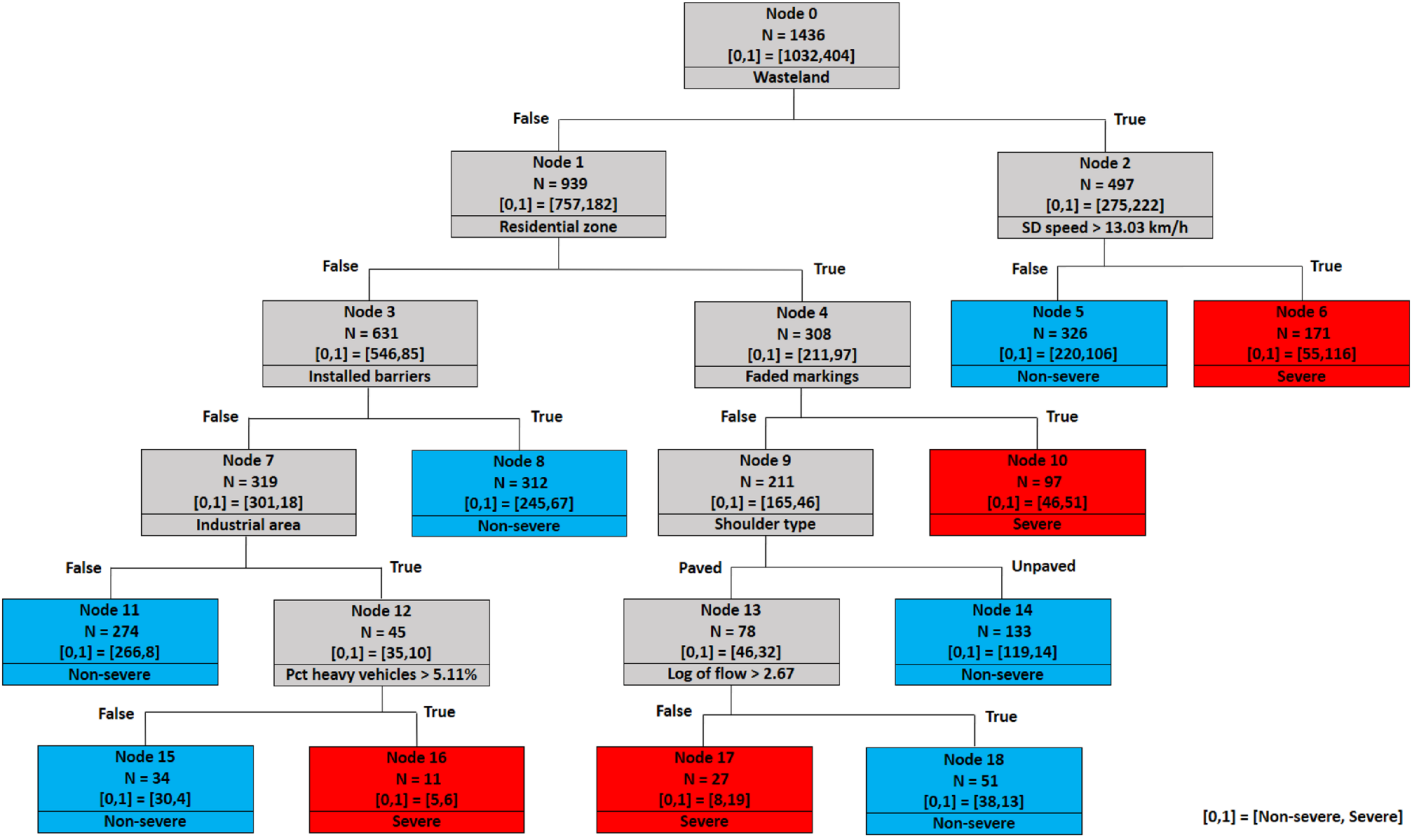
Decision tree of 3000 m spacing scenario.

Figure 10 shows the decision tree of the last scenario, including the farthest crashes from the cameras. Similar to previous models, the wasteland variable still is the first splitter. Among leaf nodes generated by the decision tree in Fig. 10, nodes 5, 6, 8, 10, 14, 17, and 18 have the same decision paths as the nodes 5, 6, 8, 10, 14, 15, and 16 in Fig. 9, respectively. The remaining terminal nodes (nodes 11, 15, and 16) reveal a new rule based on the interactive effect of industrial land use and the percentage of heavy vehicles. It was found that crashes are more likely linked to injury and fatal outcomes when heavy vehicles are involved^11^. The extracted new rule from the tree indicates that traffic compositions consist of heavy vehicles percentages greater than 5.11% in industrial districts are more prone to cause severe crashes.

As seen in the 2000 and 3000 m tree, having a greater spacing brings more non-traffic variables, such as shoulder type or industrial area, but then adds the traffic flow or percent of trucks on the subsequent node. It means that additional traffic-related variables will emerge in posterior nodes when spacing increases, which generally makes them less significant than other splitters.

### Variable importance measure (VIM)

**Figure 11:**
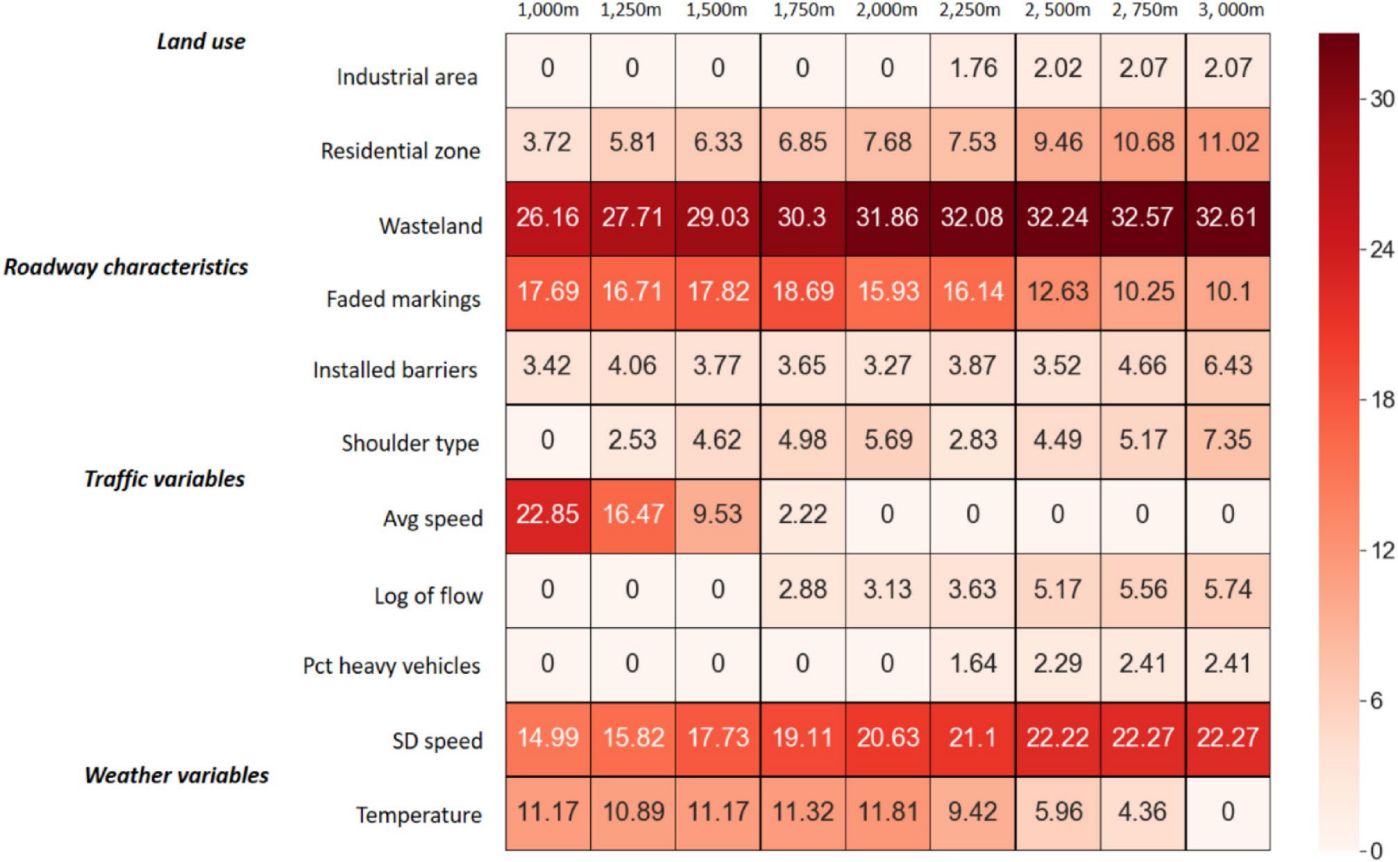
Variable importance Heat map.

The standardized VIM calculated for the models is shown in Fig. 11. It is reported in a way that its summation for all independent variables is one hundred. The dummy variable representing wastelands is the most important in all models. Studying developed trees reveals that about 43% of crashes on segments surrounded by wastelands are severe. The prevalence of severe crashes in these segments is not surprising because the speed is usually high when the road is located in the wasteland.

Not surprisingly, 1000 m model has the highest overall importance for traffic variables (sum = 37.84), containing average speed and speed deviation as significant traffic variables with a remarkable VIM of 22.85 and 14.99, respectively. However, other scenarios, which consist of more distant crashes, represent traffic variables with lower overall VIMs than the variables in 1000 m model. Moreover, the average speed is a relevant factor in 1000–1750 m range, experiencing a decline in this range and then losing its importance for the subsequent scenarios. The result is sensible because the speed at the camera will be similar to the speed at the crash if the distance is relatively small. The gradual disappearance of average speed is accompanied by the appearance of other traffic variables such as the logarithmic transformation of flow and percentage of trucks as better predictors for larger coverage scenarios. Unlike mentioned traffic variables, standard deviation of speed shows its constant presence irrespective of different spacings. Similarly, previous crash severity studies with varying layouts of detector and spacings unanimously demonstrated this variable as an influential factor in crash severity^8–10^.

Land use factors gain more importance with the increase in spacings in Fig. 11. This rise is explainable because these factors could substitute the vanishing traffic variable, average speed, as wasteland areas experience higher speeds than residential zones. Also, these factors could help better explain other traffic variables. As mentioned in the tree model of 3000 m, the percentage of trucks is determinant in industrial areas. Likewise, both variables appeared and gained importance from 2250 m scenario.

Overall, comparing presented models reveals that fluctuations in the performance of models are much less than changes in the VIMs, which implies that spacing has a substantial impact on the significance of variables rather than the performance of models.

As shown in Fig. 11, installed barriers, faded markings, residential zone, wasteland and standard deviation of speed are common variables for all the models. These variables generate three dominant rules associated with severe outcomes:

1. As stated in previous studies, high variations of speed increase the risk of severe crashes^8–10^. It was shown that high standard deviations of speed are more prone to severe crashes on wasteland roads. Enhanced enforcement for speeding would be an efficient measure to lower the risk of severe crashes in these segments.
2. In all models, faded markings increase the likelihood of severe outcomes in residential areas. Unfortunately, the maintenance of rural roads is seriously neglected in Iran, and as a result, rural residential regions require regular maintenance of markings.
3. Proper traffic barriers are installed as safety treatments to prevent serious injuries and fatalities^49^. In this research, all of the trees confirm that installing safety barriers decreases the probability of severe crashes.

## Conclusion

This research explores the relationship between crash severity of rural roads and different factors obtained from multiple data sources. In addition to variables sourced from the crash dataset, base maps, weather stations, and traffic cameras were employed to generate an integrated database. This combined database was fed into the decision tree model to detect mechanisms underlying crash severities. As cameras are fixed at their location, the distance between crash locations and cameras varies over a wide range. Concerning this issue, twelve spacing scenarios from 250 to 3000 m were introduced at first. The predictive power of the presented models was assessed based on overall accuracy and F_2_ measure. It was demonstrated that with a sufficient number of instances, different spacing scenarios succeeded in predicting severity labels. Irrespective of some differences in results, the CART algorithm revealed that wasteland, installed barriers, faded markings, residential zone indicators, and standard deviation of speed are common and important variables in the selected scenarios. According to the aforementioned variables, three pervasive rules associated with severe outcomes emerged from the models. It was found that speed variations higher than 12.3–13.1 km/h in wastelands increase the probability of severe crashes. Also, all the models demonstrated that faded markings in roads passing through residential areas and lack of safety barriers are associated with severe outcomes. Additionally, the VIMs were calculated for all independent variables. Comparing the VIMs reveals that the overall importance of traffic-related variables declines at larger distances. Moreover, Unlike average speed, flow and percentage of heavy vehicles were selected by CART as relevant traffic variables when spacing is relatively large. All in all, it was shown that spacing affects the importance of variables rather than the performance of models.

This study has several limitations: (1) Conclusions of this study are limited to the rural highways with low curvature in flat terrain, where the camera layout has an average spacing of 5.52 km. Other networks with significantly lower or higher densities of detectors may need local adaption. (2) Future studies could develop models with more severity levels and high class imbalance considering fatal severity as a distinct outcome. Moreover, the paper does not focus on modeling crash frequency but only on the crash severity. Future research could include estimating crash frequency as well. (3) The main purpose of this study is to introduce spacing scenarios and measure their effect on the real-time crash severity model. Thus, the modeling method remained unchanged while various scenarios were examined. It would be desirable to employ other techniques in the future. (4) some short coverage scenarios in this study suffer from insufficient sample size; future studies can expand their analysis period and acquire more accident reports, especially for these scenarios.

## Data Availability

The datasets generated during and/or analyzed during the current study are available from the corresponding author on reasonable request.
